# [11C]CS1P1 PET links T-cell–associated immune activation with endothelial and astrocytic responses

**DOI:** 10.21203/rs.3.rs-9237372/v1

**Published:** 2026-04-19

**Authors:** Tammie Benzinger, Savannah Tiemann Powles, David Hoagey, Shaney Flores, Karl Friedrichsen, Shuang Wu, Jayashree Rajamanickam, Nelly Joseph-Mathurin, Sarah Keefe, Sydney Nagy, Nicole McKay, Julie Wisch, Edita Sabaredzovic, Gengsheng Chen, Ashlee Simmons, Lynne Jones, Anil Kumar Soda, Jonathan Powles, Stephanie Doering, Nayid Jana, Parinaz Massoumzadeh, Bryce Baker, Carlos Cruchaga, Suzanne Schindler, Laura Ibanez, John Morris, Hongyu An, Jingxia Liu, Zhude Tu, Matthew Brier

**Affiliations:** Washington University in St. Louis; Washington University in St. Louis School of Medicine; Washington University in St. Louis School of Medicine; Washington University in St. Louis; Washington University in St. Louis School of Medicine; Washington University in St. Louis School of Medicine; Washington University in St. Louis School of Medicine; Washington University in St. Louis School of Medicine; Washington University School of Medicine - in St. Louis; Washington University in St. Louis School of Medicine; Washington University School of Medicine; Washington University Medical School; Washington University in St. Louis School of Medicine; Knight Alzheimer’s Disease Research Center, Washington University, St Louis, MO; Washington University in St. Louis School of Medicine; Washington University in St. Louis School of Medicine; Washington University in St. Louis School of Medicine; Washington University in St. Louis; Massachusetts General Hospital, Harvard Medical School; Washington University in St. Louis School of Medicine; Washington University School of Medicine - in St. Louis; Washington University in St. Louis School of Medicine; Washington University School of Medicine; Washington University School of Medicine; Washington University in St. Louis; Washington University School of Medicine; Washington University in St. Louis; Washington University in St Louis School of Medicine; Washington University in St. Louis School of Medicine; Washington University in St. Louis School of Medicine

## Abstract

Neuroimmune signaling across the peripheral–vascular–glial axis is increasingly recognized as a driver of both age-related brain vulnerability and the earliest stages of neurodegenerative disease, including Alzheimer disease. Evaluating this axis in vivo remains challenging due to limited neuroinflammatory imaging biomarkers. We utilized [11C]CS1P1 positron emission tomography (PET) to quantify sphingosine-1-phosphate receptor 1 (S1PR1) availability alongside plasma proteomics in 42 cognitively normal individuals (age 21–82). Through differential abundance analysis and structural equation modeling (SEM), we identified a multi-compartment neuroimmune cascade linking peripheral T-cell activation (CD40LG), vascular endothelial disruption (ICAM1/TEK), central S1PR1 upregulation, and reactive astrogliosis (GFAP). Mediation analysis estimated this S1PR1 axis accounts for 25.5% of the total effect of CD40LG on GFAP. This cascade appears coupled to the astrocytic immune response and is exacerbated by underlying amyloid-beta pathology. These findings suggest [11C]CS1P1 may serve as an in vivo tool for evaluating peripheral-to-central immune crosstalk.

## Introduction

Neuroinflammation is increasingly recognized as a driving mechanism of age-related neurodegeneration, spanning conditions such as Parkinson disease, frontotemporal dementia, and Alzheimer disease (AD).^1^ Peripheral inflammation plays a key role in age-related neuroinflammation and early neurodegenerative vulnerability.^2^ Peripheral immune mediators, particularly CD4+ T-cells, interact with the blood-brain barrier (BBB) endothelium to drive CNS-directed inflammatory signaling.^1,2^ One well-characterized molecular pathway underlying this process involves T-cell upregulation of CD40 ligand (CD40LG), which promotes endothelial dysfunction through adhesion molecule expression (ICAM1) and disruption of vascular stability signaling (TEK, encoding TIE2), collectively promoting BBB permeability.^3,4^ Tracking this cascade *in vivo* could enable patient stratification for anti-inflammatory trials and monitoring of therapeutic target engagement in pre-symptomatic neurodegeneration.

While plasma and CSF biomarkers can index central and peripheral inflammation independently, no tissue-level imaging tool currently captures mechanisms underlying the neurovascular immune axis *in vivo*.^5,6^ Current PET radiotracers for neuroinflammation, most notably those targeting the translocator protein 18kDa (TSPO), face significant methodological limitations.^5^ TSPO tracers are confounded by a functional polymorphism (rs6971) that stratifies individuals into high-, mixed-, and low-affinity binders, introducing inter-individual variability and complicating group comparisons.^6^

[^11^C]CS1P1, a PET radioligand developed to bind sphingosine-1-phosphate receptor 1 (S1PR1), lacks this polymorphic limitation and may address this gap.^7–9^ In the periphery, S1PR1 governs lymphocyte egress from lymphoid organs and is the primary target of fingolimod and siponimod, functional antagonists approved for the treatment of multiple sclerosis.^10,11^ At the neurovascular interface, S1PR1 signaling maintains endothelial integrity, and its disruption promotes vascular permeability and peripheral immune infiltration.^12^ During neuroinflammation, S1PR1 is upregulated on reactive astrocytes, serving as a proxy for astrocytic immune activation.^11^ Reactive astrogliosis, measured with glial fibrillary acidic protein (GFAP), and microglial activation, measured with triggering receptor expressed on myeloid cells 1 and 2 (TREM1 and TREM2), represent principal glial responses to neuroinflammatory stimuli.^13^

In this study, we pair [^11^C]CS1P1 PET imaging with high-sensitivity plasma proteomics to map the S1PR1-mediated inflammatory cascade in a cohort of lifespan cognitively normal adults with a spectrum of underlying AD pathology, indexed by plasma Aβ42/40.^14,15^ Through structural equation modeling (SEM), we evaluate the signaling pathway from peripheral T-cell activation to central astrogliosis through S1PR1. We demonstrate that [^11^C]CS1P1 captures an astrocytic-associated neuroimmune response, providing an *in vivo* framework that, unlike existing neuroinflammatory biomarkers, links peripheral-to-central immune signaling in aging and pre-symptomatic neurodegeneration. To our knowledge, this represents the first *in vivo* characterization of the full peripheral–vascular–glial neuroimmune axis at the tissue-level using a single molecular imaging tool in humans.

We evaluated 42 cognitively normal individuals (Clinical Dementia Rating^®^ (CDR^®^)=0). Baseline pathology was determined with [^11^C]PiB PET or NULISAseq plasma ptau217.^14,16^ All procedures were approved by the Institutional Review Board (IRB) and the University’s Radioactive Drug Research Committee (RDRC). [^11^C]CS1P1 dynamic PET was acquired for 90 minutes on the Siemens Biograph Vision PET/CT. Cortical standard uptake value ratios (SUVRs; 30–60 minutes post-injection) were normalized to the corpus callosum, selected based on between-subject discriminability and test-retest reliability (Supplementary Table 1, Supplementary Figure 1). Plasma proteins were quantified using the Alamar NULISAseq CNS 120 Panel v2.^14^ To identify the immune signature associated with [^11^C]CS1P1, we performed a differential abundance analysis using multivariable linear regression, adjusting for age, sex, and Aβ42/40 ratio and report uncorrected significant proteins. The hypothesized cascade was evaluated using a multi-compartment structural equation modeling (SEM).

### Cortical [11C]CS1P1 uptake is associated with central reactive astrogliosis.

To determine if in vivo S1PR1 expression reflects the astrocytic immune response, we performed multivariable linear regression. [^11^C]CS1P1 SUVR was positively associated with plasma GFAP after adjusting for age, sex, and amyloid-beta pathology (plasma Aβ42/40 ratio; β=0.128±0.055, p=0.025) (Figure 1B). This relationship was similarly robust in unadjusted rank correlations (ρ=0.401, p=0.008). Notably, TREM1, which amplifies acute inflammation, was significantly associated with [^11^C]CS1P1 uptake (β=0.169±0.067, p=0.015), while TREM2, a negative regulator of inflammation, was not (β=0.017, p=0.733), consistent with the acute inflammatory characterization of the S1PR1-associated cascade.^11,17^ These data indicate that cortical [^11^C]CS1P1 SUVR is closely related to astrogliosis, independent of baseline demographic factors or underlying AD pathology (Figure 1B).^15^

### [11C]CS1P1 captures a T-cell-associated neurovascular cascade.

**Figure 2:**
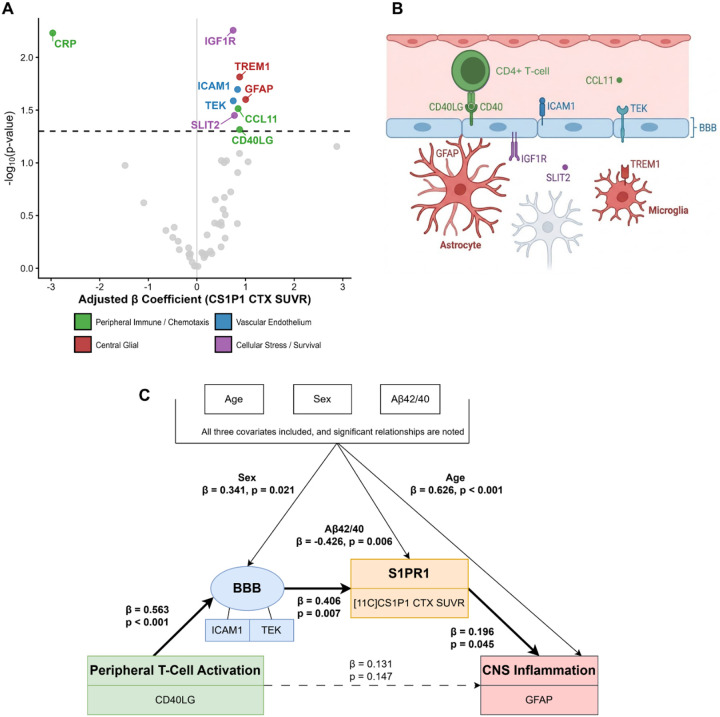
Identification and structural modeling of a multicompartment neuroimmune S1PR1 cascade. (A) Volcano plot of the differential abundance analysis evaluating 52 inflammatory proteins against cortical [^11^C]CS1P1 SUVR, controlling for age, sex, and Aβ42/40, with colors showing only the significantly associated proteins (uncorrected p<0.05). Color-coding shows established functional compartments within the neurovascular axis. (B) Biological schematic illustrating the anatomical localization of the upregulated protein signature across the blood-brain barrier (BBB) and central parenchyma. (C) Structural Equation Modeling (SEM) path diagram isolating the primary astrocytic signaling axis. Directional variance flows from CD40LG through latent BBB disruption (ICAM1/TEK) and [^11^C]CS1P1 to central astrogliosis (GFAP). Values represent standardized β coefficients.

Differential abundance analysis of 52 inflammatory proteins against cortical [^11^C]CS1P1 SUVR (Figure 2A) revealed associations with markers of peripheral T-cell activation and chemotaxis (CD40LG, CCL11), vascular endothelial dysfunction (ICAM1, TEK), and central glial activation (GFAP, TREM1; Figure 2B). In plasma, both ICAM1 and TEK reflect soluble shed forms released during endothelial activation; elevated plasma TEK indicates loss of membrane-bound TIE2 stabilizing signaling, consistent with endothelial activation.^4,18^ [^11^C]CS1P1 was also inversely associated with CRP, which was not incorporated into the neurovascular cascade. To evaluate the underlying mechanism, we constructed a multicompartment SEM, specifying the cascade direction based on established experimental evidence (Figure 2C, Supplementary Table 2).^1–3^ The primary SEM identified the following sequential cascade: peripheral T-cell activation (CD40LG) predicts blood-brain barrier disruption (ICAM1/TEK; β=0.563, p<0.001), which predicts increased cortical [^11^C]CS1P1 SUVR (β=0.406, p=0.007), and culminates in reactive astrogliosis (GFAP; β=0.196, p=0.045). Alternative models either failed to fit the data or showed worse fit (AIC=160.40, Supplementary Table 3), indicating the data best aligned with the framework depicted in Figure 2C. Plasma Aβ42/40 independently predicted [^11^C]CS1P1 uptake (β=−0.426, p=0.006, Supplementary Figure 3). The neuroimmune cascade remained significant when utilizing plasma ptau-217 as an alternative covariate (Supplementary Figure 4).

### S1PR1 availability, measured with [11C]CS1P1, links peripheral and astrocytic immune signaling.

The indirect effect estimates that this specific [^11^C]CS1P1-mediated neurovascular pathway accounts for 25.5% of the total inflammatory variance between peripheral CD40LG and central GFAP (Supplementary Figure 2A-B). Competing models evaluated TREM1 in isolation, TREM1 as a divergent dual-outcome, or TREM1 as collapsed into a single latent CNS variable with GFAP, and all failed absolute fit criteria (CFI < 0.95, RMSEA > 0.08; Supplementary Table 3). This indicates that the [^11^C]CS1P1 inflammatory cascade is coupled to the astrocytic, rather than microglial, immune response.

Here, we demonstrate that [^11^C]CS1P1 PET imaging captures a neuroimmune cascade linking T-cell-derived CD40LG, BBB integrity markers, S1PR1 availability, and reactive astrogliosis. It is well established that peripheral T-cells disrupt BBB integrity, which is associated with CNS neuroinflammation,^1–3^ and these data indicate that S1PR1 availability is a marker of this process, bridging BBB disruption to central astrogliosis. By imaging a coordinated T-cell–endothelial–astrocytic inflammatory axis, [^11^C]CS1P1 PET provides a window into neuroimmune processes that shape aging and early neurodegenerative risk.^1,2^

A notable finding of this study is the in vivo evidence demonstrating the S1PR1-axis mediates the relationship between peripheral and central inflammatory signaling via its association with reactive astrogliosis (GFAP), but not microglial activation (TREM1). Given the potential importance of this selective mediation, future research employing CSF or brain-specific microglial tracers is essential to determine the role of microglia in this signaling cascade.

These findings carry translational relevance. Fingolimod and siponimod, S1PR1 modulators approved for multiple sclerosis, demonstrate that S1PR1 is a pharmacologically accessible target, though their effects in AD models have been mixed, potentially reflecting non-selective modulation of both peripheral and central S1PR1 signaling.^11,19,20^ Among individuals with underlying AD pathology, a lower plasma Aβ42/40 ratio was significantly associated with increased cortical [^11^C]CS1P1 uptake (β=−0.426, p=0.006), suggesting this neuroimmune pathway is further amplified in the context of AD-related pathology. By capturing T-cell-associated peripheral activation together with astrocytic inflammatory responses, [^11^C]CS1P1 PET may help identify individuals with engaged neuroimmune pathways, which is an important consideration for future immune-targeted- therapeutic strategies for multiple neurodegenerative diseases.

As a hypothesis-driven pilot exploring S1PR1’s role bridging peripheral-to-central neuroinflammation, this study carries inherent limitations, though our approach remains grounded in literature. Our primary SEM sequence was chosen based on established mechanistic relationships; however, the cross-sectional design precludes definitive claims of temporal causality. A reversed model (GFAP → S1PR1 → BBB → CD40LG) fit equally well (ΔAIC < 0.2; Supplementary Table 3). This equivalence likely reflects a bidirectional neuroimmune feedback loop, highlighting the need for longitudinal evaluations to elucidate exact temporal dynamics. Additionally, while NULISAseq plasma Aβ42/40 successfully captured pathology-driven variance, future cohorts should utilize mass-spectrometry assays to more precisely measure underlying amyloid burden. Further, the modest sample size necessitated targeted feature selection and restricted statistical power. Consequently, differential abundance findings did not survive false discovery rate (FDR) correction despite large effect sizes (Supplementary Figure 2A, 2C). Therefore, individual biomarker associations warrant cautious interpretation until replicated. While these constraints emphasize careful interpretation, the strong alignment of our findings with existing literature provides a robust foundation for the large-scale validations essential to map this complex system.

These preliminary findings indicate that for the first time in humans, [^11^C]CS1P1 PET captures acascade across peripheral T-cell signaling (CD40LG), vascular endothelial markers (ICAM1, TEK), and astrocytic inflammation (GFAP). S1PR1-PET imaging may serve as an *in vivo* marker of neuroimmune interactions relevant to normal aging and AD risk.

## Online Methods

### Recruitment:

Twelve young healthy controls (age 21–37) were recruited from a research participation registry, and thirty older healthy controls (age 40–82) were recruited from the Knight Alzheimer’s Disease Research Center (ADRC) at Washington University in St. Louis. All participants in the Knight ADRC have a Clinical Dementia Rating (CDR) and Aβ-PET scan. Only participants who were cognitively normal (CDR=0) were evaluated. We aimed to recruit approximately 50% of each sex, but the final cohort is predominantly female (83% female). The inclusion criteria for this study were: male or female of any race and capable of providing written informed consent or having a legally authorized representative provide informed consent.

### Demographics Analysis:

Cohort demographics are shown in Table 1. We consider age, race, ethnicity, body mass index (BMI), white matter hyperintensities (WMH) volume, and amyloid status (Table 1). Age, sex, and Aβ42/40 were used as covariates in the differential abundance and SEM analyses.

### Blood Collection:

Up to 10 whole blood samples (~ 2 mL) were collected in heparinized syringes at sporadic intervals beginning 5 minutes post-injection. A venous blood curve was measured from blood samples obtained using a second venous line (IV in the contralateral arm, opposite the side of the injection). Additional whole blood samples, also beginning ~ 5 min post-injection were collected for HPLC radio-metabolite analysis and were taken at up to 5 time points after injection. Aliquots of whole blood for the venous curve were counted on a gamma counter, then centrifuged to obtain an aliquot of plasma that was counted to determine the plasma curve. Heparinized blood for metabolite analysis (~ 8 mL) was centrifuged to separate plasma from the blood cells. Plasma was analyzed using an automatic column-switching HPLC system which enabled the injection of large volumes (2–5 mL) of plasma onto a trapping column, the concentrated radioactivity is then eluted onto an analytical column and peaks are measured using a sensitive inline radioactivity detector to generate the radioactive chromatograph, which provided the percentage of each radioactive peak eluted from the HPLC column. Free fraction was estimated using pre scan blood and phosphate buffered saline (PBS) incubated with the remaining tracer following the standard ultrafiltration technique for analysis of plasma samples loaded onto Millipore centrifuge units.

### MRI and PET Acquisition:

MRI was acquired on a Siemens Biograph mMR hybrid PET/MR scanner at 3T field strength. Only older participants underwent a dynamic amyloid PiBPET scan concurrent with MRI acquisition on the Biograph mMR. Structural MRI scans included a T1-weighted magnetization-prepared rapid gradient echo (MPRAGE) and a T2-weighted fluid-attenuated inversion recovery (FLAIR) sequence at 1mm isotropic resolution in accordance with the ADNI4 protocol. [^11^C]CS1P1 was produced in-house and injected at a dose of 12 mCi (+/−2.4) followed by a saline flush at the start of a 0–90 minute dynamic PET acquisition on the Siemens Biograph Vision PET/CT.

### MRI and PET Processing:

Structural T1 images were segmented into cortical and subcortical regions of interest (ROIs) with Freesurfer 7.1.1, and visually inspected for quality control by trained imaging processors following FreeSurfer-recommended guidelines. PET scans were pre-processed using the PET Unified Pipeline (PUP), which includes scanner resolution harmonization at a full-width half-maximum of 8mm, rigid registration between frames to correct for motion, rigid registration to the processed structural T1-weighted MRI, and summing the frames covering the 30–60 minute post-injection window for both [^11^C]PiB and [^11^C]CS1P1. The T2 FLAIR scans were processed through the TrUE-Net pipeline to calculate total white matter hyperintensity volume, which was then log_10_-transformed as it followed a log-normal distribution.

Centiloid, a measurement of widespread cortical amyloid uptake, is determined using the MR-Free Pipeline on the PiB-PET, and we used a positivity threshold of 20 Centiloids.^14^ For [^11^C]CS1P1, we derived cortical and white matter SUVRs by first generating a voxelwise, body-weighted standard uptake value (SUV) image from the summed 30–60 minute image. Using the FreeSurfer segmentations of the structural T1-weighted MRI, we combined all cortical gray matter regions into a composite cortical ROI and all white matter regions into a composite white matter ROI. With FMRIB Software Library (FSL)’s *fslstats* tool, we extracted the mean SUVs for both composite ROIs, as well as the corpus callosum. Composite SUVs were then divided by the SUV of the reference region to create their respective SUVRs using *fslmaths*.

### Protomics:

Plasma was thawed, centrifuged to separate plasma, and 25 μL was used for the NULISAseq CNS Disease Panel 120 (version 2). Relative protein abundance is reported in NULISA Protein Quantification (NPQ) units, which are log2-transformed values derived from raw sequencing data. To ensure accuracy, the raw reads undergo a multi-step normalization process. First, intra-plate variability is corrected by normalizing the raw count of each analyte against the Internal Control (IC) count within that specific sample. Next, to account for batch effects across different plates, these values are adjusted relative to the median signal of three Inter-plate Controls (IPCs) included on each plate. Finally, the data is rescaled and log2-transformed to generate NPQ values, resulting in an approximately normal distribution suitable for statistical analysis. Cut point analysis determined the ptau-217 threshold of 11.8 NPQ. The covariate of continuous plasma Aβ42/40 ratio was measured with this panel. NULISAseq uses a robust quality control method and provides the user with highly sensitive and specific protein measurements.^13^

### Statistical Approach

#### Feature Selection and Differential Abundance:

To identify the peripheral and central proteomic signature associated with cortical S1PR1 expression, a differential abundance analysis was performed on 52 neuroinflammatory-designated proteins from the NULISAseq CNS Disease 120 panel v2. The neuroinflammatory-designated proteins were prioritized as we aim to understand the specific inflammatory profile the tracer captures and have a modest sample size, limiting the power of this analysis. Separate multivariable linear regression models evaluated the association between each individual protein and cortical [^11^C]CS1P1 standardized uptake value ratio (SUVR), adjusting for age, sex, and continuous plasma Aβ42/40 ratio, as it accounts for more variance than binary amyloid status. Proteins were considered significant at an uncorrected threshold of p < 0.05 to enable targeted feature selection for downstream structural modeling. Significant proteins were subsequently categorized into functional biological compartments (e.g., peripheral immune/chemotaxis, vascular endothelium, central glial), and their inter-relationships were assessed and visualized using a hierarchically clustered Spearman’s rank correlation matrix.

#### Structural Equation Modeling (SEM):

To evaluate the mechanistic cascade linking peripheral immunity to central neuroinflammation, Structural Equation Modeling (SEM) was performed using the lavaan package in R. The primary model tested a sequential neuroimmune axis: peripheral T-cell activation (CD40LG) to blood-brain barrier (BBB) disruption to S1PR1 activation ([^11^C]CS1P1 SUVR) to central astrogliosis (GFAP). To account for measurement error and biologically group the endothelial response, BBB integrity was modeled as a latent variable (an unobserved construct estimated from ICAM1 and TEK jointly). Age, sex, and Aβ42/40 ratio were included as direct covariates on all endogenous variables. Aβ42/40 is a necessary covariate in this analysis as it accounts for inflammatory variance driven by amyloid pathology (Supplementary Figure 3). Plasma Aβ42/40 was utilized as a continuous covariate rather than a binary classification to preserve statistical power and capture sub-threshold variance in amyloid accumulation. Binarizing continuous variables is known to reduce power, which is important given the cohort’s modest sample size and low number of amyloid-positive individuals (n=5). A sensitivity analysis confirmed the loss of statistical power when utilizing a binarized amyloid covariate. Models were fit using standard Maximum Likelihood (ML) estimation to ensure covariance matrix stability given the cohort sample size (N = 42). Absolute model fit was evaluated using the Comparative Fit Index (CFI; ideal ≥ 0.95), Root Mean Square Error of Approximation (RMSEA; ideal < 0.08), and the model χ^2^ p-value (ideal > 0.05).

#### Mediation Analysis and Specificity Testing:

The formal mediation effect (indirect effect) of the S1PR1-specific pathway was calculated as the product of the structural path coefficients and expressed as a proportion of the total effect of CD40LG on GFAP. To test the specificity of the neurovascular cascade signal, alternative structural configurations integrating microglial activation (TREM1) were evaluated. These included single-outcome (TREM1 only), dual-outcome (divergent paths to separate GFAP and TREM1 nodes), and combined Latent CNS (GFAP and TREM1 as interchangeable indicators) models. Competing models utilizing identical baseline covariance matrices were formally compared using relative parsimony indices, specifically the Akaike Information Criterion (AIC) and Bayesian Information Criterion (BIC), where lower values indicate superior model fit. All statistical analyses were performed in R (version 4.5.2), with statistical significance defined as p < 0.05.

## Supplementary Material

Supplementary Files

This is a list of supplementary files associated with this preprint. Click to download.

- SubmissionCode.zip
- SupplementarySPowles.pdf
